# Cardinal Points in the Successful Insertion of Permanent Fillings

**Published:** 1898-10

**Authors:** H. C. Kahlo

**Affiliations:** Indianapolis, Ind.


### AMERICAN JOURNAL

—OF—

### DENTAL SCIENCE.

VOL. XXXII. BALTIMORE, OCTOBER, 1898. NO. 6.

### Selections.

ARTICLE I.

### CARDINAL POINTS IN THE SUCCESSFUL INSERTION OF PERMANENT FILLINGS.

DR. H. C. KAHLO, INDIANAPOLIS, IND.

I have selected for this paper a very familiar subject; one which has so frequently been brought before societies that I fear it may be classed in the category of "has beens;" yet I will venture the assertion that few have reaped, to the fullest extent, the harvest which it contains.

Unfortunately, I haven't in my possession a rule, or set of rules, which, if followed, will insure success in every case, for the conditions present, and the complications that arise, are too manifold to admit of that. To be able to master all of these difficulties at all times would mean the attainment of a degree of perfection such as, I fear, none have reached.

If this be correct, let us continue the pursuit along this line so long as there still remain points which severally tax our skill as operators. If we will consider ourselves as students, for such we are, or should be, we cannot but realize that it is the constant reviewing of these familiar subjects which enables us to reach, or at least approach, that degree of perfection to which we aspire. For convenience sake, I have subdivided the subject as follows:

Preparation of cavity-walls.
Removal of decay.
Establishment of anchorage.
Use of matrices.
Finishing of fillings.

There are a number of other items which come rightly within the province of this paper, such as the advantages of separation, choice of filling materials, exclusion of moisture, etc., all of which are important but somewhat accessory, especially as we are considering fillings in a general sense.

The preparation of cavity-walls would naturally come first, since the opening up of a cavity is the first thing which occupies our attention.

In reality, it was this particular feature upon which must fall the blame for the writing of this paper. It may be an injustice to burden cavity-walls with more than their share, yet I feel no sense of charity toward that which I consider the bitterest and most persistent enemy I have had to encounter in my efforts to reach a degree of proficiency in my chosen profession.

In the "American Text-Book on Operative Dentistry" Dr. Guilford has contributed a very comprehensive and interesting chapter on this subject. He shows us by illustrations that the enamel rods radiate outwardly on all convex surfaces of the teeth, and inwardly—that is to say, toward the dentine—on all concave surfaces. This is a most valuable point to be borne in mind in the preparation of cavity-walls. If unheeded, the result will almost invarably be fracture and subsequent dissolution of these enamel-rods, and, as a consequence, reappearance of decay.

Another feature is the necessity of having these cavity margins sustained by dentine. Many of us have too often fallen into error in the preparation of cavity margins by the all-absorbing interest in the retention of our fillings, failing to trim away the margin sufficiently for fear of destroying the natural undercuts which the walls present. If experience has taught me one lesson more thoroughly than any other in the practice of dentistry, it is the desirability of having thick, well-sustained cavity margins. Cavities in the approximal surfaces of bicuspids and molars should be freely opened up in this manner for the additional reason that by so doing we cut beyond the points of contact with the adjacent tooth, and thereby spare ourselves the annoyance of the re-establishment of decay, which would otherwise often occur at these points.

Generally speaking, my aim is to have the cavity-walls as nearly parallel as possible, radiating outwardly at an angle of 15 degrees, on all approximal surfaces and perpendicular on masticating surfaces. In most cases these margins can be perfectly prepared by the use of sharp chisels.

We all realize the importance of removing all signs of decay from within cavities so far as is practicable for the safety of the underlying nerve, so I shall pass on immediately to another and more fruitful source of failure.

Assuming that we have faithfully carried out the first step, it is imperative that we exercise our best judgment in the selection of the points at which we desire to establish anchorage.

Two or three things must be taken into consideration, viz:

First. We must have anchorage as near as possible in the line of greatest resistance.

Second. We must guard against the weakening of the maagins.

Third. We must not destroy or impair the source of nutriment.

It is evident therefore, that it is much easier to tell where not to place undercuts than where to place them.

Fortunately, nature comes to our rescue in a great many cases, in the natural anchorage which cavities present. Of the others, we can usually decide upon a means if we are mindful of the dangers such as have been stated. Personally, I am particularly opposed to the custom, resorted to more in the past than in the present, namely, the cutting of deep pits or a deep groove along the cervical surface of cavities. It usually is extremely painful to the patient and certainly greatly interferes with the normal nutrition of the tooth crown. Let me assert here the desirability of the common use of matrices.

Many cavities are naturally undercut, but without the use of a matrix, great difficulty is experienced in the starting of fillings and the building of them up to a point where the natural undercuts exist. In all such cases, especially those in distal surfaces of teeth, I use a matrix (usually a Guilford matrix), but before securing it in position I place one or two pellets of gold along the cervical margin, allowing them to overhang so that when the matrix is pushed up into position and clamped, these pellets are firmly secured. In this way I have a perfect starting-point for my filling, in addition to the assurance that my cervical margin is perfectly covered. Where it can be done, it is a good practice to flatten a large pellet which will cover the cervix and side walls both, binding it in place with the matrix.

In these cases I use pellets of semi-cohesive gold, unannealed at first, but annealing after the foundation of metal has been laid.

I have learned to place a great deal of reliance upon firm and adequate anchorage in the masticating surfaces of all teeth which contain approximal cavities of a size which justify the opening to that surface, and nearly all do. With a natural anchorage in the approximal surface and a strong artificial one, upon the masticating surface, there is little chance of failure from this cause.

Another advantage in the use of matrices is that they dispense with a great deal of the annoyance and labor incident to finishing.

Where gold is thoroughly malleted against the wall of a matrix which is secure, and afterwards burnished, it requires very little finishing with strips and discs. Nor do I mean to confine the utility of matrices in connection with gold as a filling material. In the filling of large approximal cavities with amalgam, their application is entirely satisfactory in enabling us to pack our material more solidly and in giving it a desirable contour. In such cavities, too, especially in devitalized teeth, we often find considerable difficulty in obtaining sufficient anchorage.

Where I consider it more practicable to fill than to crown, I nearly always resort to a very convenient set of anchor-screw appliances made by the S. S. White Manufacturing Company.

By their use it is possible to establish, in a few moments, an anchorage in one or two roots such as will enable you to build a surprisingly large and secure filling over.

The last, but by no means the least, important function in the correct insertion of permanent fillings is the finishing.

Here I want to lay special emphasis upon thorough burnishing, claiming for it hardness of surface and fineness of finish in direct proportion to the thoroughness of its application. It is a very difficult matter to fill a cavity, especially with gold, so that all the particles along its surface are firmly and perfectly welded together.

Burnishing enables us to perfect where the mallet has failed, especially along the cavity margins. My custom is to fill cavities a little more than full, allowing for a certain amount of condensation as the result of burnishing. Following this comes the use of approximal trimmers, strips and discs, until the filling is reduced to a point where it is flush with the cavity margins.

As a precautionary measure, the points of union between cavity margin and filling should be gone over with an explorer to assure ourselves that there are no overhanging edges.

Now, with the use of tape or polishing points, the operation is completed.

There seems to be a natural inspiration for operators to take pains in the finishing of gold fillings. Whether it is the color, the fee, or the pride which lies in the knowledge that they are working in precious metals, I do not know, but I do know that too many disregard the imporance of finishing amalgam fillings. If we expect permanency in them, they should be finished with the same amount of care we give to gold fillings. If we can't afford to do it in consideration of the fee, let us charge more or put a lower estimate upon the value of our time.

It has been said that the average life of permanent fillings is three years.

Of course, many failures cannot consistently be laid at the door of our profession, but I am sure patience, care, and perhaps a little more conscience on the part of the dental profession generally will insure a remarkable decrease in this mortality rate.—*Indiana Dental Journal.*

